# Multidrug Resistance in Cancer Circumvented Using a Cytosolic Drug Reservoir

**DOI:** 10.1002/advs.201700289

**Published:** 2017-11-09

**Authors:** Li Fan, Silu Zhang, Chunyuan Zhang, Chun Yin, Zhiqin Chu, Chaojun Song, Ge Lin, Quan Li

**Affiliations:** ^1^ Department of Pharmaceutical Analysis The Fourth Military Medical University 169th Changle west road Xi'an Shaanxi 710032 China; ^2^ Department of Physics The Chinese University of Hong Kong Shatin New Territories Hong Kong; ^3^ Beijing Computational Science Research Center No.10 East Xibeiwang Road Haidian District Beijing 100193 China; ^4^ School of Biomedical Sciences The Chinese University of Hong Kong Shatin New Territories Hong Kong; ^5^ Department of Immunology The Fourth Military Medical University 169th Changlewest road Xi'an Shaanxi 710032 China

**Keywords:** circumventing multidrug resistance, cytosolic drug concentration, cytosolic drug release, optical switches, self‐decomposable nanoparticles

## Abstract

It is discovered that sustained cytosolic drug release at a sufficient concentration is an effective mechanism to circumvent multidrug resistance and consequently enhance antitumor drug efficacy. It is showed that a simple way to enable this mechanism is to reach an intracellular kinetic balance of the drug movement between the drug released from the carrier into the cytosol and the one removed from the cell interior. By adopting nanoparticle (NP) as the drug carrier, a reservoir of drug can be maintained inside the cells upon effective cellular uptake of these NPs via endocytosis. This study shows that gradual release of the drug from the NP carrier provides a feasible scheme for sustained drug release in cells, resulting in relatively stable cytosolic drug concentration level, particularly in the drug resistant case. By implementing an “optical switch” with light irradiation on photosensitizer in the same nanoparticle carrier, cytosolic drug release is further promoted, which increases cytosolic drug concentration with good concentration retention. Enhanced drug efficacy in drug sensitive as well as resistant models is demonstrated both in vitro and in vivo. Such a mechanism is shown to efficiently circumvent multidrug resistance, and at the same time largely reduce the systemic toxicity of the anticancer drug.

## 1 Introduction

Multidrug resistance (MDR) developed in many cancer cells is a crucial issue limiting the efficacy of cancer chemotherapeutics. MDR can develop from a variety of mechanisms,^1^ and one of the most common mechanisms involves the efflux of drugs from cells by adenosine 5′‐triphosphate‐binding cassette (ABC) transporters, such as P‐glycoprotein (P‐gp) and multidrug resistance protein 1. The resultant insufficient intracellular concentration of drug leads to the reduced drug efficacy. Consequently, a high dose is required to ensure the sufficient therapeutic level, but also leads to more severe adverse effects and high systemic toxicity.

Common methods to avoid ABC transporter‐mediated MDR included direct inhibition of the transporter protein and/or using nucleic acids (e.g., siRNA) to knock down genes involved in MDR. However, MDR might develop via multiple pathways, and the overall effects of the transport proteins and relevant genes on cell physiology remained unclear. The uncertainties led to unsuccessful application of the inhibitors or siRNA on clinical therapies. In fact, therapies that target one MDR pathway were found to enhance the ability of cancer cells to adapt and develop other drug resistance pathways such as alternating efflux transporters.^2^, ^3^, ^4^, ^5^, ^6^, ^7^

Recently, nanocarrier‐drugs have been proposed as promising candidates against the MDR effect, as being mainly ascribed to their easy cellular uptake, i.e., cellular entry of “disguised” drugs via nanoparticle (NP) endocytosis would lead to enhanced cellular uptake of drugs.^8^, ^9^, ^10^, ^11^, ^12^, ^13^, ^14^, ^15^, ^16^ Although enhanced drug efficacy was indeed observed in some cases, whether there is a MDR reversal effect remained unclear, because of the lack of comparisons of drug efficacy between drug sensitive cells and its corresponding resistant cells. More importantly, possible underlying mechanism(s) of nanocarriers assisting the drug bypassing the MDR was unrevealed, the understanding of which would otherwise provide a novel approach for design of drug MDR reversal effect.

In the present work, we disclosed an effective mechanism to circumvent the MDR effect, being responsible for the significantly enhanced drug efficacy. Using pairs of Human breast cancer cell lines MDA LCC6 versus its P‐gp‐overexpressed subline MDA LCC6 MDR1, and Human ovarian carcinoma cell lines Hela versus its P‐gp‐overexpressed subline ADR‐Hela for the direct comparison, we demonstrated the restored activity of anticancer drug in the resistant cancer cells using the a self‐decomposable silicon dioxide (methylene blue)‐doxorubicin (SiO_2_(MB)‐Dox) NP carrier drug system. The similar IC_50_ values of the selected anticancer drug Dox observed in both parental and resistant cell lines suggested the complete circumvention of MDR in the resistant cells. By further decorating the surface of SiO_2_(MB)‐Dox NPs with polyethylene glycol (PEG) and folate acid (FA) and injecting the NP carrier drugs in mice bearing Hela and ADR‐Hela xenografts, we achieved remarkably enhanced drug efficacy with significantly reduced systemic toxicity in vivo. Mechanistically, we disclosed that upon cellular uptake, the NPs served as the reservoir of Dox, and the sustained release of Dox from the self‐decomposable NPs provided a stable intracellular concentration. With further aid of the “optical trigger,” significantly enhanced drug efficacy was achieved in both drug sensitive and resistant cells. In the present work, the photosensitizer MB served as the “optical trigger,” controlled optical excitation of which promoted the cytosolic escape of Dox molecules by increasing the permeability of the endo/lysosome membrane,^17^, ^23^, ^24^ and thus increased the drug concentration in the cytosol. The sustained release of drug at adequate and stable cytosolic drug concentrations provided a feasible scheme to circumvent the MDR effect.

## 2 Results and Discussions

### 2.1 Characterizations of SiO_2_(MB)‐Dox NPs

**Figure 1:**
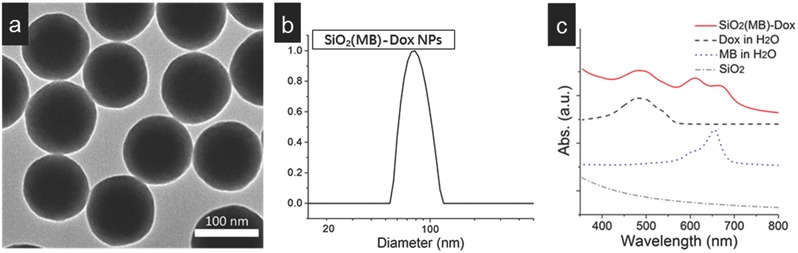
Morphology and composition of the SiO_2_(MB)‐Dox NPs. a) Typical TEM image of the SiO_2_(MB)‐Dox NPs, the scale bar is 100 nm; DLS data of SiO_2_(MB)‐Dox NPs. b) Hydrodynamic diameter of the NPs measured by DLS. c) Optical absorption spectra of SiO_2_(MB)‐Dox NPs, pure Dox, and MB in aqueous solutions, and pure SiO_2_ NPs.

The morphology of the SiO_2_(MB)‐Dox NPs is shown in **Figure** **1**a, which is a typical transmission electron microscopy (TEM) image. They were spherical in shape with an average diameter of ≈80 nm (Figure 1a). This is consistent with the hydrodynamic diameter measured from ensembles of the same nanoparticles by dynamic light scattering (DLS), as shown in Figure 1b. Successful incorporation of both MB and Dox into the SiO_2_ NPs was suggested by the optical absorption spectrum taken from the NP samples (Figure 1c). Pure SiO_2_ had little absorption in the visible wavelength range. By referencing to the pure MB absorption measured in aqueous solution (dotted line), the MB characteristic absorption at ≈665 and ≈600 nm observed in the NP sample (solid line) suggested the presence of MB in the sample. The relatively higher intensity of the dimer absorption peak at ≈600 nm (as compared to that of the monomer at ≈665 nm) indicated aggregation of MB molecules, when they were grown into the SiO_2_ NPs.^17^ The characteristic absorption peak of Dox at ≈490 nm (reference made to pure Dox in aqueous solution, as shown by the dashed line) was also recognized in such NP samples, indicating the successful loading of Dox into the SiO_2_(MB) NP carrier.

Both Dox and MB were found to be released from the NPs (dispersed in 50% serum) (Figure S1, Supporting Information). A relatively fast escape of Dox molecules from NPs was observed in the first 24 h, followed by a gradual slow release phase. The release of MB was slightly different from that of Dox. A rather slow release profile in the first 12 h was observed, followed by a boost in the next 12 h, and eventually leveled off after 48 h. This was mainly due to the fact that Dox was absorbed onto the surface and subsurface layer of the NPs, while MB was mostly concentrated in the center of NPs.^18^

Carrier self‐decomposition is an important feature of such NPs. The corresponding morphological evolution of the SiO_2_(MB)‐Dox NPs can be found in Figure S2a–d (Supporting Information). The decomposition details can be found in previous reports.^18^ It was known to be triggered by the diffusion driven MB release from the center of the NPs, and complete carrier decomposition would be reached when all of the drugs were released.^18^ The specific drug release and carrier decomposition pattern are determined by the structural characteristic of the NP, that is, the MB is highly concentrated in the center of the NPs and a loose SiO_2_ network entangles with MB in the NP.^18^

### 2.2 In Vitro Study of Cellular Uptake, Release, and Efficacy of the NP‐Drug

#### 2.2.1 Enhanced Cellular Uptake of Dox via NP Carrier

**Figure 2:**
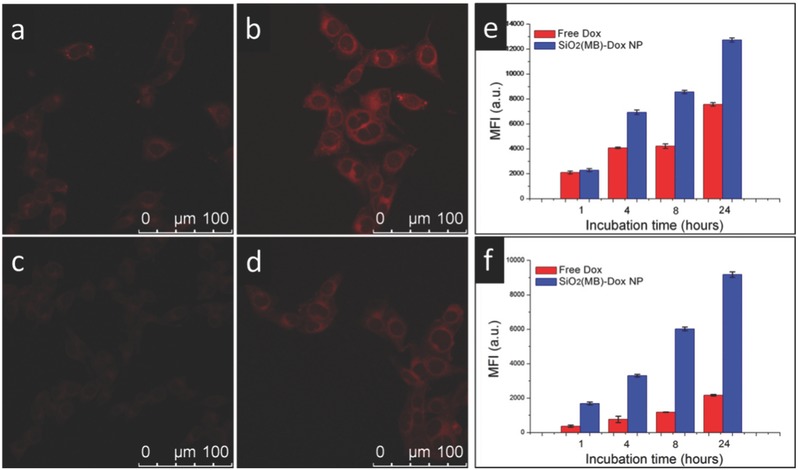
Cellular uptake comparison of free Dox and Dox loaded in NP carrier at the same feeding concentration (of Dox). Confocal images showing MDA LCC6 cells after incubation with a) free Dox, or b) SiO_2_(MB)‐Dox NPs for 24 h. Confocal images showing MDA LCC6 MDR1 cells after incubation with c) free Dox, or SiO_2_(MB)‐Dox NPs for 24 h. d) Fluorescence signal of Dox in the confocal images was represented by red color. Flow cytometry results show the time dependent total Dox accumulation in e) MDA LCC6 cells and f) MDA LCC6 MDR1 cells.

The cellular uptake of Dox in the free drug form or in the SiO_2_‐MB NP carrier was first evaluated using confocal microscopy. Here we considered the total cellular uptake amount of Dox regardless of their forms present in the cells (e.g., residing in the NPs or being released to cytosol). The uptake of Dox was suggested by its fluorescence signal in both MDA LCC6 cells and MDA LCC6 MDR1 cells, after incubation with free Dox or SiO_2_(MB)‐Dox NPs for 24 h (**Figure** **2**a–d). We observed that the fluorescence intensities of pure Dox in both MDA LCC6 (Figure 2a) and MDA LCC6 MDR1 (Figure 2c) cells were weak, and the signal from the resistant cells was hardly detectable. As a comparison, the respective fluorescence intensity of Dox (loaded in SiO_2_(MB)‐Dox NPs) was much stronger in both cell lines, although the signal was consistently weaker in MDA LCC6 MDR1 (Figure 2d) than in MDA LCC6 cells (Figure 2b). Quantitative results were obtained using flow cytometry with different incubation time (Figure 2e,f). The results were consistent with the confocal observation. In addition, we found that Dox fluorescence intensity in both cell lines gradually increased along with the incubation time. Higher Dox fluorescence intensity was always found in SiO_2_(MB)‐Dox NPs treated cells, as compared to free Dox treated ones. Such difference was more significant in MDA LCC6 MDR1 cells (Figure 2f).

The free Dox is hydrophilic and enters cells mainly via passive diffusion.^19^, ^20^ The NPs enter the cells via endocytosis,^18^, ^21^ which is a more effective pathway for carrying Dox into the cells. The more remarkable difference in the cellular uptake amount between free Dox and SiO_2_(MB)‐Dox NPs observed in the resistant MDA LCC6 MDR1 cells suggested that additional benefit of incorporating Dox into the NP carrier—Dox in the nanoparticle carrier when entering the cells, the so called “disguise” effect. Nevertheless, one should note that the total cellular uptake amount of Dox, including those being released to cytosol and those not being released yet, did not directly correlate to the cytosolic concentration of Dox, which is the most critical factor in determining drug therapeutic efficacy.

#### 2.2.2 Release Strategy to Improve the Cytosolic Escape of Drug Molecules

Although NPs carrier enhanced the cellular uptake of drug molecules, these NPs resided in membrane bounded vesicles upon their entry into the cells, as a result of endocytosis (Figure S3, Supporting Information). Therefore, cytosolic release of drugs (represented by the drug concentration in the cytosol) required drug molecules not only leaving the NP carrier but also escaping from the vesicle compartments, the passive escape of which led to rather low cytosolic drug level.

To tackle this problem, we needed a mechanism that helped the drug molecules to escape from the vesicle compartments (lysosomes in the present case), once they were released from the NP carrier. MB has been widely used as a drug of photodynamic therapy, based on the mechanism that MB generates ^1^O_2_ within cells, and the generation of reactive oxygen species leads to apoptotic cell death.^22^ Nevertheless, at controlled concentration and light irradiation conditions, MB can produce a small amount of reactive oxygen species that leads to the increased invesicle membrane permeability^17^, ^23^, ^24^ but has little effect on cell viability (Figure S4, Supporting Information). Here we showed that by incorporating MB in the NP carrier, one could manipulate the membrane permeability of the vesicles containing the NPs, but caused little cytotoxic effect. This was realized by controlling a low concentration of MB (concentration below 5 × 10^−6^ m) and short light irradiation duration (within 5 min). Details on the determination of these parameters are elaborated in Figure S4 (Supporting Information).

The escape of Dox from the vesicle compartment (lysosomes here) and its cytosolic release were investigated by confocal microscopy. After the cells were incubated with SiO_2_(MB)‐Dox NPs for 24 h without light irradiation, one can see that concentrated Dox fluorescence signal in a randomly spotted pattern in the cells (Figure S5b, Supporting Information) coinciding well with the lysosome signal (Figure S5a, Supporting Information). This suggested that most of Dox molecules were confined inside the lysosomes, rather than being released to cytosol. As a comparison, after the cells were exposed to light irradiation for 5 min (determination of the irradiation duration can be found in the Supporting information), a rather diffusive Dox fluorescence background was observed (Figure S5d, Supporting Information) compared with lysosome signal (Figure S5c, Supporting Information), with much less intensive fluorescence spots, suggesting a more efficient escape of Dox molecules to the cytosol.

**Figure 3:**
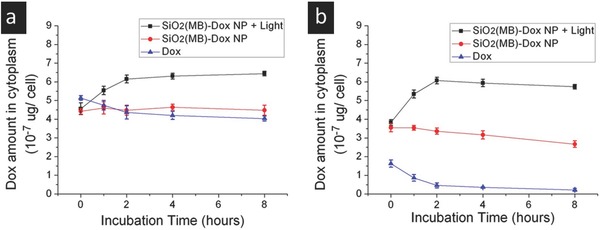
Disposition of Dox in the cytosol of cells. Dox amount in the cytosol of a) MDA LCC6 cells and b) MDA LCC6 MDR1 cells along with the incubation time in Dox‐free culture medium after preincubation with free Dox, or SiO_2_(MB)‐Dox NPs, or SiO_2_(MB)‐Dox NPs followed by light irradiation.

We then examined the concentration change of Dox in the cytosol of both MDA LCC6 and MDA LCC6 MDR1 cells, after their being incubated with SiO_2_(MB)‐Dox NPs or free Dox for 24 h and then transferred to a drug free medium for further incubation (defined as the starting time point in **Figure** **3**). Statistical analysis of the data can be found in Figure S6 (Supporting Information).

In MDA LCC6 cells, Dox concentration in cytosol was found to be similar (≈4–5 × 10^−7^ µg per cell) in both SiO_2_(MB)‐Dox NPs and free Dox fed cells at all time points examined. Nonetheless, a gentle decreasing trend of Dox concentration was observed in free Dox fed cells, while a stable Dox concentration was obtained in SiO_2_(MB)‐Dox NPs fed ones. For the same NP fed cells, a significant increase in Dox concentration was detected in the cytosol upon light irradiation, before a stable concentration was eventually reached, being 1.37 times of that without light irradiation (Figure 3a).

More drastic difference in the cytosolic Dox concentration among the three groups (i.e., free Dox fed cells, SiO_2_(MB)‐Dox NPs fed cells with and without light irradiation) was observed in MDA LCC6 MDR1 cells. In Figure 3b, one can see that the cytosolic Dox concentration was low in free Dox fed cells (1.63 × 10^−7^ µg per cell), and quickly dropped to much lower values—after only 2 h incubation, it was measured at 0.46 × 10^−7^ µg per cell, which contiunously dropped to even lower value with longer incubation time. A decreasing trend was also observed in the SiO_2_(MB)‐Dox NPs fed cells, but much more gently as compared to the case of free Dox. Despite the decreasing trend of Dox cytosolic concentration measured in the NPs treated MDA LCC6 MDR1 cells, the concentration range (3.55–2.66 × 10^−7^ µg per cell) was not significantly lower than that measured in MDA LCC6 cells (≈4.50 × 10^−7^ µg per cell) with other conditions kept the same. Upon the assistance of light irradiation, one can see a surge of Dox concentration in cytosol within the first two hours (reaching ≈6.07 × 10^−7^ µg per cell). This value was similar to that observed in MDA LCC6 cells at the same time point. A gentle decrease in Dox concentration then followed, but did not significantly affect the level of Dox concentration (remained at ≈5.73 × 10^−7^ µg per cell after 8 h incubation).

In the free Dox treated MDA LCC6 and MDA LCC6 MDR1 cells at all time points examined, significant differences in the intracellular Dox concentration were observed (*P* < 0.0001), indicating the MDR in MDA LCC6 MDR1 cells (Figure S6a, Supporting Information). In the case of free Dox fed MDA LCC6 cells, its concentration decrease in the cytosol was mainly resulted from diffusion‐driven exclusion due to the Dox concentration difference inside and outside cells. This explained the decreasing trend of Dox concentration in MDA LCC6 cells in the first two hours (Figure 3a). On the other hand, MDA LCC6 MDR1 cells overexpressed with P‐gp,^25^ leading to efficient efflux of Dox out of cells. Consistently, one observed both the significantly reduced cellular uptake of free Dox (Figure 2f) in MDA LCC6 MDR1 cells, and its significant concentration decrease with further incubation in drug free medium (Figure 3b) for the first two hours. In both cell lines, the obvious intial drop (within 2 h) of the intracellular Dox concentration followed by slightly further decrease, revealed the intial exclusion of Dox from cells before a concentration equilibrium was reached.

The incorporation of the NP‐carrier enabled a different cellular uptake pathway for Dox, i.e., endocytosis, a direct consequence of which is the residence of NPs (loaded with drugs) in membrane bounded vesicles.^[21,18]^ Although the spontaneous Dox desorption allowed the release of Dox from the NP carrier, most of them remained in the vesicle compartments. Their cytosolic release was only realized when Dox (desorbed from the NP carrier) diffused through the vesicle membrane to cytosol. This explained the similar cytosolic Dox concentration observed in free Dox and NPs fed MDA LCC6 cells (Figure 3a, blue and red lines), despite the difference in their total Dox cellular uptake amount (Figure 2e). On the other hand, we found stable and gently decreasing cytosolic Dox concentration in NP‐fed (Figure 3a, red line) and free Dox‐fed (Figure 3a, blue line) MDA LCC6 cells, respectively. The stable cytosolic Dox level in the case of NP fed cells indicated the kinetic balance of Dox movement between leaving NP carrier/vesicle compartments and its passive diffusing from cells (diffusion‐driven exclusion). In this regard, the NP carrier drug was advantageous to free Dox as it maintained at a relative stable cytosolic drug concentration. Nevertheless, one may expect no significant drug efficacy enhancement in the present MDA LCC6 cells, as the Dox cytosolic concentrations for free drug and SiO_2_(MB)‐Dox drug were similar. This was indeed observed in the cell viability test, as discussed in later sections.

When MDA LCC6 MDR1 cells were employed, the significantly increased cytosolic concentration of Dox in the case of SiO_2_(MB)‐Dox NPs (vs free Dox) initially (time point 0 in Figure 3b) originated from increased total cellular uptake amount of Dox (Figure 2f). As Dox molecules were stored in the SiO_2_(MB)‐Dox NPs and being gradually released to the cytosol, it provided a relatively stable cytosolic Dox concentration even though efflux persisted, which was revealed by the slow Dox cocentration decrease (Figure 3b, red line) in the resistant cells (but not in the parental cells).

The idea of incorporating the optical switch (MB molecules that can increase vesicle membrane permeability upon light irradiation) was to enhance Dox diffusion through the membrane of the vesicle compartment, and thus the cytosolic concentration of Dox. The experimental observation of cytosolic Dox concentration increased in the first two hours in both MDA LCC6 (Figure 3a, black line) and MDA LCC6 MDR1 cells (Figure 3b, black line) confirmed the vesicle confinement of Dox (after their being released from the NP carrier), and the effective “open‐up” of the vesicle compartment for Dox escape to cytosol upon light irradiation. Eventually (after the initial 2 h), a kinetic balance of the Dox disposition was reached in both cell lines, as suggested by the sustained (Figure 3a) and slight concentration decrease (Figure 3b) of the drug in the drug sensitive and resistance cells, respectively. The experimental result showed that turning on the optical switch significantly increased the cytosolic Dox concentration by 1–2 times in both cell lines. The insignificant difference (Figure S6b,c, Supporting Information) of the intracellular Dox concentration (upon light irradiation) in the drug sensitive and resistant cell lines suggested MDR reversal in the latter.

#### 2.2.3 Enhanced Dox Efficacy in Multidrug Resistant Cancer Cells

**Figure 4:**
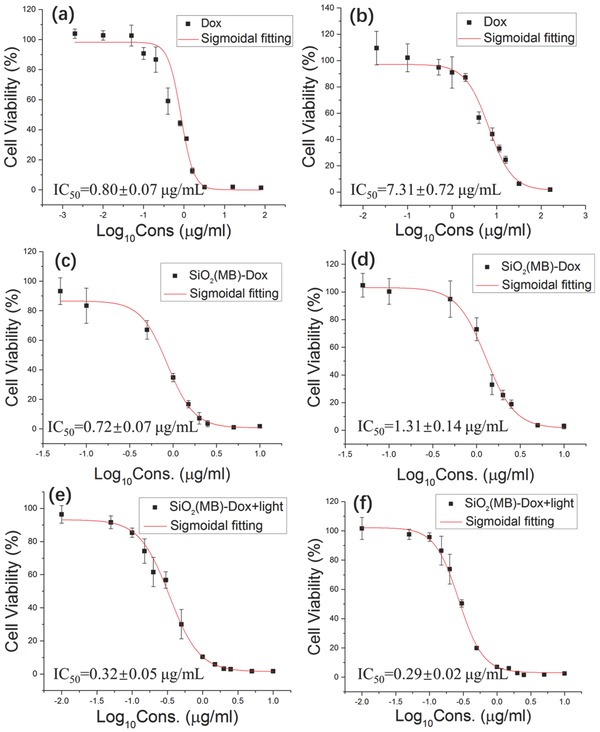
The viability of a,c,e) MDA LCC6 cells and b,d,f) MDA LCC6 MDR1 cells after exposure to a,b) pure Dox; c,d) SiO_2_(MB)‐Dox NPs; and e,f) SiO_2_(MB)‐Dox NPs with 5 min light irradiation. Data are presented as mean ± standard deviation (SD) (from three independent experiments) and significantly different (*p* < 0.05) from control (analyzed by Student's *t*‐test).

The drug efficacy of free Dox and SiO_2_(MB)‐Dox NPs in MDA LCC6 cells and MDA LCC6 MDR1 cells was investigated by measuring cell viability using 3‐(4,5‐dimethylthiazol‐2‐yl)‐2,5‐diphenyltetrazolium bromide (MTT) assay (**Figure** **4**). Statistical analysis of the data can be found in Figure S7a (Supporting Information).

We first looked at the cytotoxicity of free Dox in both MDA LCC6 and MDA LCC6 MDR1 cells in response to different drug concentrations (Figure 4a,b). The Dox showed obvious cytotoxicity in MDA LCC6 cells at low concentrations, the half maximal inhibitory concentration (IC_50_) was estimated as 0.80 ± 0.07 µg mL^−1^. While in MDA LCC6 MDR1 cells, the cytotoxicity of Dox was significantly lower, and the IC_50_ was estimated as 7.31 ± 0.72 µg mL^−1^, being an order of magnitude larger than that in MDA LCC6 cells. The significant differences in the IC_50_ values measured in the two cell lines (Figure S7a, Supporting Information) suggested MDR effects in MDA LCC6 MDR1 cells, i.e., the overexpressed P‐gp actively pumped Dox out of the drug resistant cells. As a result, one needed to largely increase the Dox dose in order to achieve the same cytotoxicity.

When Dox was loaded into SiO_2_(MB) NP carrier, the drug efficacy was about the same as free Dox in MDA LCC6 cells (Figure 4a vs 4c; Figure S7a, Supporting Information). This was expected as one recalled the similar cytosolic Dox concentration in the two corresponding cases. In addition, this observation also suggested that the enhanced total drug cellular uptake itself (Figure 2e) not necessarily led to drug efficacy enhancement, while it was the available cytosolic drug concentration that determined the drug efficacy. When such an experiment was carried out in MDA LCC6 MDR1 cells (Figure 4b vs 4d; Figure S7a, Supporting Information), the drug efficacy enhancement (as compared to free Dox) was found to be significant. The IC_50_ was decreased to 1.31 ± 0.14 µg mL^−1^ (Figure 4d), being ≈6 times lower than that of free Dox (7.31 ± 0.72 µg mL^−1^ in Figure 4b). In fact, the difference in IC_50_ values of Dox (loaded in the SiO_2_(MB) NPs) in MDA LCC6 MDR1 and MDA LCC6 cells was no longer significant, suggesting reversal of the MDR in the drug resistant cells.

The difference in IC_50_ values of Dox (loaded in the SiO_2_(MB) NPs) in MDA LCC6 MDR1 and MDA LCC6 cells completely disappeared when the cytosolic Dox concentration was increased by turning on the optical switch (enhanced vesicle membrane permeability for easy Dox diffusion to cytosol and thus increased cytosolic drug concentration), indicating complete reveral of the MDR in the drug resistant cells. The Dox activity was enhanced in both MDA LCC6 and MDA LCC6 MDR1 cells upon light irradiation. The IC_50_ of Dox in MDA LCC6 and MDA LCC6 MDR1 cells were further decreased to 0.32 ± 0.05 and 0.29 ± 0.02 µg mL^−1^, respectively (Figure 4e,f). Both values were two times lower than the IC_50_ of free Dox in MDA LCC6 cells. These results demonstrated the dual effects of MDR reversal and Dox activity enhancement by employing NP carrier drug with optical switch.

The MTT results showed excellent correlation with the measured cytosolic concentration profile of Dox (Figure 3). The observed drug efficacy enhancement by employing SiO_2_(MB)‐Dox NPs resulted from their unique cytosolic Dox concentration profiles in both cell lines, i.e., the increased drug concentration and retention in cytosol were responsible for the enhanced Dox efficacy.

MTT assays were also conducted on Hela and ADR‐Hela cell lines using free Dox and MB(Dox)‐SiO_2_‐FA NPs (with/without irradiation), before the in vivo test. Similar trends of IC_50_ in the respective cell lines were observed as those in MDA LCC6/MDA LCC6 MDR1 cell lines (Figures S7b and S8, Supporting Information).

### 2.3 In Vivo Evaluation of the NP‐Drug System

Effective drug accumulation at the tumor site is a prerequisite for NP based drug delivery in vivo. In this regard, folate‐conjugated nanoparticles were commonly employed, as their folate grafting would help them actively and specifically target to cancer cells.^26^, ^27^ On the other hand, folate grafted to PEGylated cyanoacrylate nanoparticles was found to have a tenfold higher apparent affinity to the folate‐binding protein than free folate.^28^ Therefore, we decorated PEG–FA on the NPs surface to achieve “active targeting” by receptor‐mediated cell internalization, taking advantage of the overexpression of folate receptors on the surface of Hela and ADR‐Hela cells. The successful decoration of PEG–FA on the NPs surface was demonstrated by both Fourier Transform infrared spectroscopy (FTIR) characterizations of the respective nanoparticles (Figure S9, Supporting Information) as well as the promoted cellular uptake of MB(Dox)‐SiO_2_‐FA NPs, comparing to that of MB(Dox)‐SiO_2_ NPs as shown in Figure S10 (Supporting Information). Its targeting capability was further suggested by in vivo imaging (Figure S11, Supporting Information). Enrichment of Dox at the tumor site was observed after 48 h incubation with the NP‐drug in both Hela and ADR‐Hela models alike (Figure S12, Supporting Information). PEG is known to reduce the probability of nanoparticles being engulfed by phagocytic cells and also slow down the drug release from the nanoparticle carrier by capping the pores of the nanoparticle carrier.^29^ Literature reports showed that only ≈10% drug release is obtained in the first 24 h when PEG capping is employed.^29^, ^30^ Consequently, the prolonged blood circulation of the nanoparticles (with little drug release) due to PEG coating would result in a peak accumulation of the nanoparticles at longer hours, which was found at ≈48 h in the present work, as suggested by the in vivo imaging observation of strong tumor site florescence signal and reduced fluorescence intensity elsewhere in the mice at this time point (Figure S13, Supporting Information). Only upon endocytosis of the NPs at the tumor site, the more acidic environment (including intrinsic tumor extracellular acidity (pH, 6.8), endosome/lysosomes (pH 4–5)) triggerred the drug release.^31^, ^32^ These mechanisms provided a reasonable explanation on the evolution of Dox amount at the tumor site.

**Figure 5:**
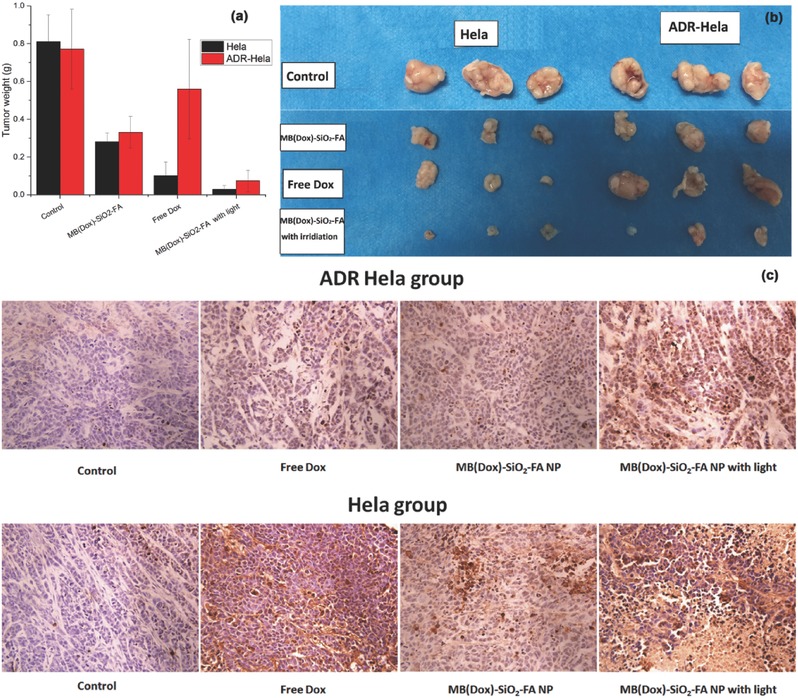
Antitumor effect of MB(Dox)‐SiO_2_‐FA NPs on nude mice bearing Hela and ADR‐Hela cells subcutaneously was studied. a) Values of tumor weight are expressed as mean ± SD (*n* = 5). b) Dissected tumor tissues from the nude mice. c) TUNEL assay of control, free drug, and NPs treatment groups with/without irradiation in Hela and ADR‐Hela tumor bearing groups.

We then evaluated the antitumor efficacy of MB(Dox)‐SiO_2_‐FA NPs in a xenograft mouse model by intravenous (i.v.) injection. The nanoparticle drug significantly delayed subcutaneous Hela and ADR‐Hela tumor growth, as demonstrated by the tumor weight evolution (**Figure** **5**a,b). In the absence of light irradiation, the efficacy of MB(Dox)‐SiO_2_‐FA NPs in treating the Hela tumor is less than that of free Dox. This may be explained by the delayed delivery of the drug to tumor. Nevertheless, significantly delayed tumor growth was achieved in the NP treated ADR‐Hela tumor, as compared to that treated by free Dox, revealing the effective circumvention of MDR mechanism via the nanoparticle drug delivery. With light irradiation, significantly delayed tumor growth in both Hela and ADR‐Hela tumor model was achieved when treated with MB(Dox)‐SiO_2_‐FA NPs, as compared to both the control and free Dox treated groups. The results are consistent with the in vitro findings. The degree of apoptosis was then evaluated by Tunel assay in both Hela and ADR‐Hela tumors. Being consistent with our in vivo antitumor efficacy results, tumors from NPs treated groups exhibited apoptosis of different levels, especially when light irradiation is simultaneously incorporated with the NPs treatment, the apoptosis signals (number of nucleusin brown colour) were of the greatest percentage (Figure 5c).

**Figure 6:**
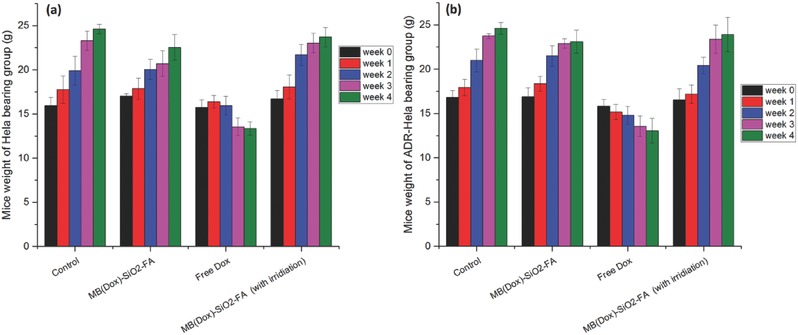
The effects of NPs treatment on nude mice bearing Hela and ADR‐Hela cells. Values of body weight changes in a) Hela and b) ADR‐Hela cells bearing mice are expressed as mean ± SD (*n* = 5). Mice were administered via i.v. injection every 5 d for 4 weeks.

Furthermore, changes in body weights were also investigated to evaluate the systemic toxicity of NPs treatment groups in both drug sensitive and resistant models. The results can be found in **Figure** **6** and Figure S14 (Supporting Information), and the statistic analysis of the data at week 4 was shown in Figure S15 (Supporting Information). Comparing to the control groups, free Dox treatment significantly reduced the body weight of mice after 4 weeks, indicating potential toxicity of Dox in the applied dosage regimen in both animal models. Figure S14 (Supporting Information) also clearly shows weight loss in free Dox treated animals, which is consistent with the weight measurements (Figure 6). The normal increase in body weight in the NPs treated groups with/without irridiation was always comparable to that of control groups (Figure 6; Figure S15, Supporting Information). In addition, comparing to the control group, the NPs treated mice showed no significant organ lesions, while obvious lesions were observed in splenic germinal center and glomerulus in free Dox treatment groups (Figure S16, Supporting Information). All animals were under good survival conditions without any of them dying during the treatment. The results demonstrated that using MB(Dox)‐SiO_2_‐FA NPs as delivery systems significantly reduced the potential systemic toxicity of Dox.

## 3 Conclusion

In conclusion, we demonstrated that NP carrier drugs served as a reservoir for sustained cytosolic release of drugs (Dox in the present case), contributing to a stable intracellular drug level. Together with the installation of an “optical switch” (in the same NP carrier) that can control the cytosolic drug concentration, we achieved drug retention in the cytosol at increased drug level, leading to the complete reversal of MDR effect and the significantly enhanced drug efficacy particularly in drug resistant models. The incorporation of the “optical switch” in the NP carrier drug allowed us to separately evaluate the cytosolic drug concentration level and its retention, enabling the understanding of their respective roles in drug efficacy enhancement. The greatly reduced IC_50_ values in the resistant cell line, the significantly suppressed tumor growth in the resistant xenograft mouse model, and largely decreased systemic toxicity disclosed the important concepts of (1) “drug reservoir” at intracellular level as a feasible MDR circumventing mechanism, and (2) increased intracelluar drug release to promote drug activity, the dual effects resulted indesired efficacy with conventional dosage regimen even in the MDR cancer cells. Such a mechanism is of universal importance as it does not rely on specific modification of the cell functions to circumvent the MDR effect.

## 4 Experimental Section

*Preparation and Characterization of SiO_2_(MB)‐Dox NPs*: To obtain the SiO_2_(MB)‐Dox NPs, the self‐decomposable SiO_2_(MB) NPs were first synthesized, the synthetic methodology of which can be found elsewhere.^18^ Briefly, 2 mg MB was added to a mixture of 75 mL ethanol with 3.4 mL 25% ammonia‐water solution, then 0.08 mL Tetraethyl orthosilicate (TEOS) was added. The SiO_2_(MB) NPs were obtained after 24 h stirring, and washed several times before being dried. After that, the Dox molecules were incorporated into the SiO_2_(MB) NPs by adsorption, as the silica matrix was negatively charged, allowing for effective loading of the positively charged Dox molecules. Typically, 10 mg SiO_2_(MB) NPs were soaked in 1 mL Dox aqueous solution (2 mg mL^−1^) for 24 h. The NPs were then collected by centrifugation and washed for several times. Furthermore, in order to make the NPs obtain active targeting function, SiO_2_(MB) NPs were first surface functionalized with amino by (3‐aminopropyl)triethoxysilane, then PEG–FA was used to decorate the NPs surface by classic amide reaction. The surface modification by PEG–FA was characterized by FTIR (Figure S9, Supporting Information). Following the synthetic protocol from the PEG–FA producer (Nanocs., USA), the PEG–FA concentration on NPs was estimated as 1.5 × 10^−8^ mol mg^−1^ NP. The ratio of PEG–FA on the particles was estimated as 454 PEG–FA molecules per NP. Drug loading capacity was determined by UV–VIS spectrometer, and calculated by the formula below, Drug loading capacity =$\frac{{\mathrm{Weight}}_{\mathrm{drug}}}{{\mathrm{Weight}}_{\mathrm{drug}\;\mathrm{loaded}\;\mathrm{nanoparticles}}\;}\times 100\%$. The MB loading capacities of MB(Dox)‐SiO_2_ and MB(Dox)‐SiO_2_‐FA NPs were both about 20%, while Dox loading efficiencies were both about 28.6%.

The general morphology and the size distribution of the NPs were characterized using TEM (PhilipsCM120). All of the optical absorption spectra were acquired using Hitachi U‐3501 UV–visible–NIR spectrophotometer.

*Drug Release and Carrier Decomposition of SiO_2_(MB)‐Dox NPs in 50% Serum in Saline*: To study the release of Dox and MB from the NPs, the SiO_2_(MB)‐Dox NPs (3 mg mL^−1^) were suspended in 1 mL simulated body fluid (with 50% fetal bovine serum (FBS), 50% saline) for specified periods at 37 °C. At each time point, the supernatant was separated from NPs by filtration with a centrifugal filter (molecular weight cut off: 30 000 Da, Millipore), after that, fresh 50% serum in saline was added for further incubation. A series of supernatant were collected at different time points and their UV/Vis absorption spectra were measured using Hitachi U‐3501 UV–visible–NIR spectrophotometer. The amounts of released Dox and MB molecules were respectively determined by the spectrum peak intensities of Dox (480 nm) and MB (660 nm), which were proportionate to their concentrations.^33^ The degradation of the SiO_2_ carrier was monitored by morphology investigation using TEM at the same time points.

*Cell Culture*: The Pg‐p‐overexpressing human breast carcinoma cell line MDA435/LCC6/MDR1 and parental cell line MDA435/LCC6/WT were generous gifts from Dr. Robert Clarke (Georgetown University, Washington, DC). Hela cell line (ATCC, CRM‐CCL‐2) was kindly provided by Department of immunology, The Fourth Military Medical University. Dox resistance cell model (ADR‐Hela) was established by continuous exposure of Hela cells to increasing concentrations of Dox over a period of 6 months. The initial Dox concentration was 1 µg mL^−1^ medium; drug concentration was increased gradually in a 1.5 µg mL^−1^ step with passage period of 3 d. After reaching a final Dox concentration of 0.1 mg mL^−1^ medium, the resistant subline was compared with the parental stock sample. The cells were cultured in Dulbecco's modified Eagle's medium, supplemented with 10% heat‐inactivated FBS, 2.0 g L^−1^ sodium bicarbonate, 0.1 g L^−1^ streptomycin sulfate, 0.06 g L^−1^ penicillin G, and 5.958 g L^−1^ 4‐(2‐hydroxyethyl)‐1‐piperazineethanesulfonic acid (HEPES). The cells were maintained in a standard, cell culture incubator at 37 °C in a humidified atmosphere with 5% CO_2_.

All of the NPs were sterilized by steaming at 115 °C (NPs in powder form) for 2 h and dispersed in the medium by slight ultrasonication (5 s) right before their introduction to the cells. Cells were seeded at initial densities of 5 × 10^4^ cells mL^−1^ in dishes and incubated for 24 h before introducing NPs, after that the original NP‐free medium was discarded and the fresh prepared NP‐containing medium was added.

*Cellular Uptake of the Drug*: To qualitatively compare the cellular uptake of Dox with or without NP carriers, MDA435/LCC6/MDR1 and MDA435/LCC6/WT cells were incubated with free Dox or SiO_2_(MB)‐Dox NPs at the same Dox concentration (2 × 10^−6^ m). After incubation for 24 h, cells were carefully washed with PBS, and stained by lysotracker (LysoTracker Green DND‐26, ThermoFisher Scientific L7526) for 40 min, then observed using confocal laser scanning microscopy (TCSP5, Leica) with a 63× water‐immersion objective lens at 504 nm excitation and 511 nm emission. For quantitative comparison, cells were incubated with free Dox or SiO_2_(MB)‐Dox NPs for 1, 4, 8, 24 h. Afterward, the cells were harvested and washed with PBS for 3 times, then suspended in 1 mL PBS for analyses using flow cytometer (FACScan, Becton Dickinson, Canada).

*Drug Release and Retention in Cells*: MDA435/LCC6/MDR1 and MDA435/LCC6/WT cells were first incubated with free Dox or SiO_2_(MB)‐Dox NPs for 24 h, followed by being washed and transferred to a drug free medium. Then the cells fed with NPs were irradiated with 590 nm LED for 5 min or not, and further incubated for specified periods. To quantitatively determine the amount of Dox release to cytosol, the cells were washed by PBS and counted, after which the cells underwent lysis in PBS containing 0.1% Triton X‐100 at 37 °C for 1 min, and were centrifugated (16 000 × g) to separate the released Dox molecules (in the cytosol) from the residual NPs (mainly existing as NP aggregates in the endo/lysosomes). The supernatants containing the cytosolic released Dox molecules were collected and their fluorescence spectra were taken to quantify the concentration of the released Dox molecules. The average amount of the cytosolic released Dox per cell was calculated by dividing total Dox amount by the cell numbers.

*Cell Viability Assay*: MTT assay was conducted to evaluate the cell viability. Briefly, after incubation with a series concentration of pure Dox or NPs for 24 h, cells were transferred to a drug free medium. The NPs treated cells were irradiated with 590 nm LED for 0/5/10 min. Then all the cell samples were further incubated for 24 h. After that, cell culture medium was replaced with MTT assay solution (0.5 mg mL^−1^) and the cells were further incubated for 4 h at 37 °C. Then MTT solution was removed and the dimethylsulfoxide was added. The absorbance was measured at 570 nm with a reference of 690 nm using amicroplate reader (#680, Bio‐Rad). The relative cell viability was calculated as a percentage compared to the control samples (treated with fresh medium without NP/drug). The effect of MB dose and light irradiation (590 nm LED) duration to the cell death were also checked by MTT assay. Cells were incubated with SiO_2_‐MB carriers at a series of MB concentrations, followed by being exposed to light for various durations. The cell viability was measured by MTT assay with the same protocol.

*In Vivo Evaluation—Assessment of Antitumor Effect in Tumor Xenograft*: All animal experiments were approved by the Animal Experiment Administration Committee of the Fourth Military Medical University. The animal model was established using Hela cells, as drug targeting to Hela cells can be readily achieved using folic acid decorated nanoparticle carriers. Hela and ADR‐Hela cells (5 × 10^6^ cells, total volume 0.1 mL) were injected into mice (Female BALB/C nude mice, 4–6 weeks) leg subcutaneously to establish tumors. When the diameters of tumors were above 0.2 cm measured by callipers, the mice bearing tumors were randomly divided into 4 groups (5 mice per group), saline control, free Dox, MB(Dox)‐SiO_2_‐FA NPs, and MB(Dox)‐SiO_2_‐FA NPs with irradiation groups. Free drug or NPs dissolved in saline (with the same Dox concentration at 5 mg kg^−1^) were administered through tail intravenous (i.v.) injection every 5 d for 4 weeks. Every mice of MB(Dox)‐SiO_2_‐FA NPs with irradiation group was conducted light irridiation (590 nm LED with distance of 5 cm from the animal skin) for 5 min at 24 h after injection. Since the xenograft tumor was established subcutaneous, the light could easily penetrate the skin and reach the tumor. In the case of deep tumors, either a different photosensitizer of near‐infrared excitation should be adopted and/or optical fibre has to be introduced to the close vicinity of the tumor site. All animals were monitored for activity, physical condition, body weight, and tumor growth. Bodyweight of each mouse was measured and recorded every week until sacrifice (mice were sacrificed at day 28 after treatment). Before sacrifice, the animals got anaesthesia by intraperitoneal injection of pentobarbital sodium (16 mg mL^−1^, 0.1 mL per mice), then saline infusion. Tumor masses were removed and weighed. All of the data are reported as the means ± S.D.

*H&E and TUNEL Assay*: Tumor masses and major organs were fixed in 4% paraformaldehyde at 4 °C overnight, and embedded in paraffin for preparing 5 µm thick sections, followed by H&E (haematoxylin/eosin, Beyotime, China) and TUNEL study. Histology and morphology of tumor and major organs were observed under the Eclipse E800 microscope (Nikon, Japan). The DeadEnd Colorimetric TUNEL assay kit (Promega, Madison, WI, USA) was used to determine apoptotic cells in tumor sections, following the manufacturer's protocols. This assay measures biotinylated nucleotide incorporation in DNA, which was then visualized by Horseradish Peroxidase (HRP)‐labeled streptavidin and 3,3N‐Diaminobenzidine Tertrahydrochloride (DAB). Images were randomly taken from 400× in each tumor section.

*In Vivo Imaging*: For in vivo bioimaging, xenograft tumor mice received a tail intravenous injection of MB‐SiO_2_‐FA NPs solution (1 mg mL^−1^, 0.1 mL). The animals were fully anesthetized by inhalation of a mixture of oxygen with 5% isoflurane. The in vivo bioimages were acquired using an in vivo fluorescence imaging system (IVIS Lumina II) with excitation wavelength at 640 nm.

*Biodistribution Study*: For blood drug concentration, 1 mL blood was collected from each mice for further study. For tissue drug concentration determination, mice after anesthesia were first infused by saline to remove the NPs remaining in the blood in major organs. Then tissues were weighed and multigelation were carried out for 5 times, then homogenated at 0 °C followed by centrifugation (16 000 g) to separate the released drug molecules from the residual NPs. The supernatants were collected for drug concentration determination by fluorescence spectrophotometer at *E*_x_ = 470 nm, *E*_m_ = 594 nm, with standard curve, *Y* = 3207*X* + 1981, where *Y* represents intensity, *X* for concentrations. *R* ^2^ = 0.99948.

## Conflict of Interest

The authors declare no conflict of interest.

## Supporting information

Supplementary
