# Supplementary material for: Multidrug Resistance in Cancer Circumvented Using a Cytosolic Drug Reservoir
Source: Adv Sci (Weinh). 2017 Nov 9;5(2):1700289. doi: 10.1002/advs.201700289 (PMC5827467; doi:10.1002/advs.201700289)
Supplement: Supplementary file 1 — Supplementary [file ADVS-5-1700289-s001.pdf]

Copyright WILEY-VCH Verlag GmbH & Co. KGaA, 69469 Weinheim, Germany, 2017.

ADVANCED SCIENCE
Open Access

Supporting Information

for *Adv. Sci.,* DOI: 10.1002/advs.201700289

Multidrug Resistance in Cancer Circumvented Using a Cytosolic Drug Reservoir

*Li Fan, Silu Zhang, Chunyuan Zhang, Chun Yin, Zhiqin Chu, Chaojun Song, Ge Lin, and Quan Li**

Copyright WILEY-VCH Verlag GmbH & Co. KGaA, 69469 Weinheim, Germany, 2017.

Supporting Information

Multidrug resistance in cancer circumvented using a cytosolic drug reservoir

Li Fan[1,#], Silu Zhang[2,3,#], Chunyuan Zhang[4], Chun Yin[4], Zhiqin Chu[2], Chaojun Song[5], Ge Lin[4], Quan Li[2,]*

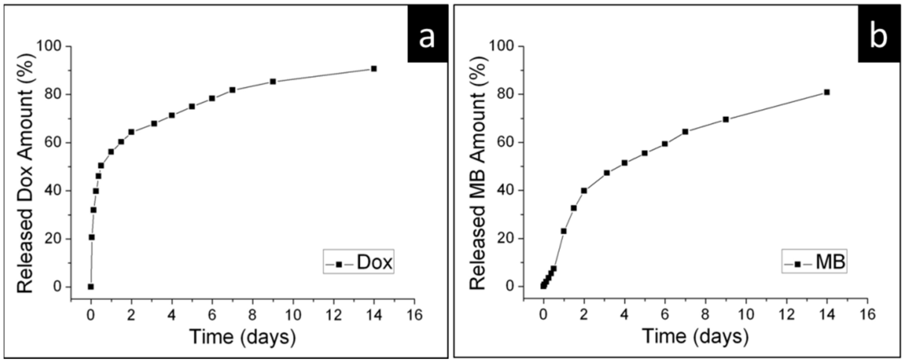

Figure S1. Release profile of Dox and MB. Evolution of the (a) Dox and (b) MB molecules being released from the NPs as a function of the immersing duration in the 50% serum in saline.

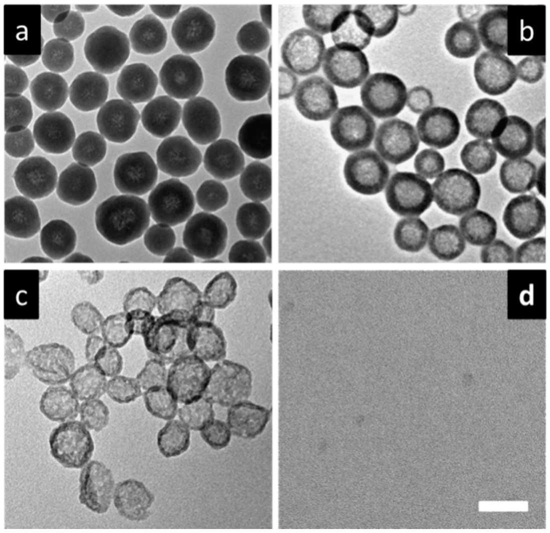

Figure S2. Decomposition of the SiO_2(MB)-Dox NPs. Typical TEM images of the NP carrier drug after their being immersed in 50% serum in saline for (a) 1 day; (b) 4 days; (c) 9 days; and (d) 14 days. The scale bar is 100 nm.

Most of the NPs remained intact at day 1, although light contrast started to appear in the NPs' center. Obvious hollow feature appeared in the center of most of the NPsafter 4 days' immersion. Such center-hollowed feature continued to enlarge in the following days, leaving a spherical shell of SiO_2 with thinner and thinner shell thickness. At day 9, some of the nanoshells appeared as porous and partially damaged, and even longer duration (14 days) led to complete collapse of the nanoshell structure to scattered fragments (**Figure S2d**).

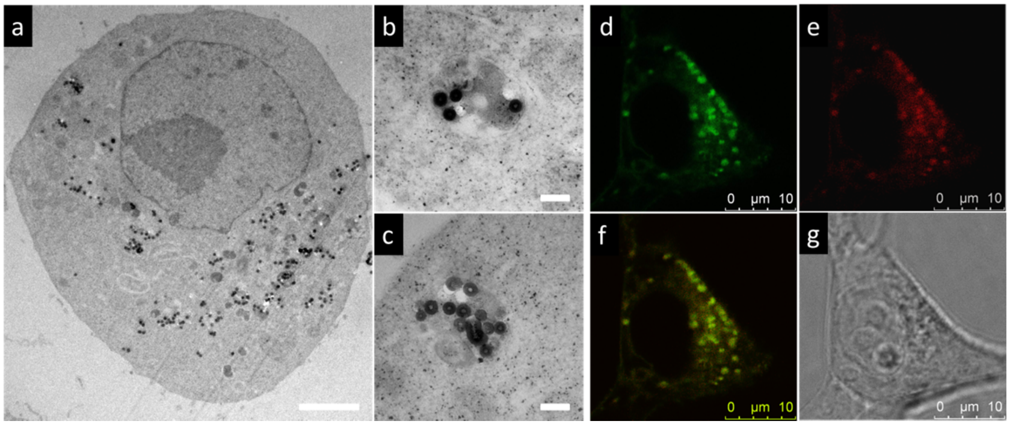

Figure S3. Intracellular distribution of SiO_2(MB)-Dox NPs. (a) TEM image showing one of the MDA LCC6 MDR1 cells after being incubated with SiO_2(MB)-Dox NPs for 24 hours, the scale bar is 2μm; (b) and (c) Typical examples showing NPs residing inside the membrane bounded vesicles, the scale bar is 200 nm. (d) Confocal microscopy images showing the intracellular location of (d) lysosomes, (e) NPs, (f) overlap of lysosomes and NPs, and (g) transmission image of the respective cell in (d), (e), and (f).

Irradiation of the SiO_2-MB carrier rendered the ROS generation. On the one hand, a small amount of ROS locally (e.g. close to the membrane bounded vesicles containing NPs) helped to increase the membrane permeability of the vesicle compartment, and thus promoted the cytosolic release of Dox when they desorbed from the NP carriers. On the other hand, large amount of ROS presence had toxic effects, eventually leading cells to apoptosis/necrosis. In our design, incorporation the photosensitizer MB is employed to increase he vesicle membrane permeability, so that we can independently evaluate the contribution of “sustained cytosolic drug release”

and “increase cytosolic drug concentration” to the final drug efficacy. Therefore, we tried to minimize MB dose and light irradiation duration, so that MB contribution to the cell death can be negligible. The appropriate parameters were identified by studying cell viability when they were incubated with SiO_2-MB carriers at various MB concentrations, followed by their being exposed to light for certain durations. Cell viability was then measured by 3-(4,5-Dimethylthiazol-2-yl)-2,5-diphenyltetrazoliumbromide (MTT) assay. As shown in **Figure S4b and c**, for both drug sensitive MDA LCC6 and drug resistant MDA LCC6 MDR1 cells, the photo-toxicity of the SiO_2-MB carrier was promoted with increase of both MB concentration and the light irradiation time. Firstly 10 min light irradiation on cells without NPs feeding did not cause much change to the cell viability in both drug sensitive and resistant cell lines (**Figure S4a**). When NPs were fed to the respective cell lines, the cell viability maintained at >90%for irradiation duration of 1 min. and 5 min at low MB concentrations (<5 μM). Based on these experiment results, we chose the irradiation time as 5 min and the MB concentration below 5 μM for further experiments, the cleaved caspase-3 test also showed these parameters were safe to the cells (**Figure S4d-f**).

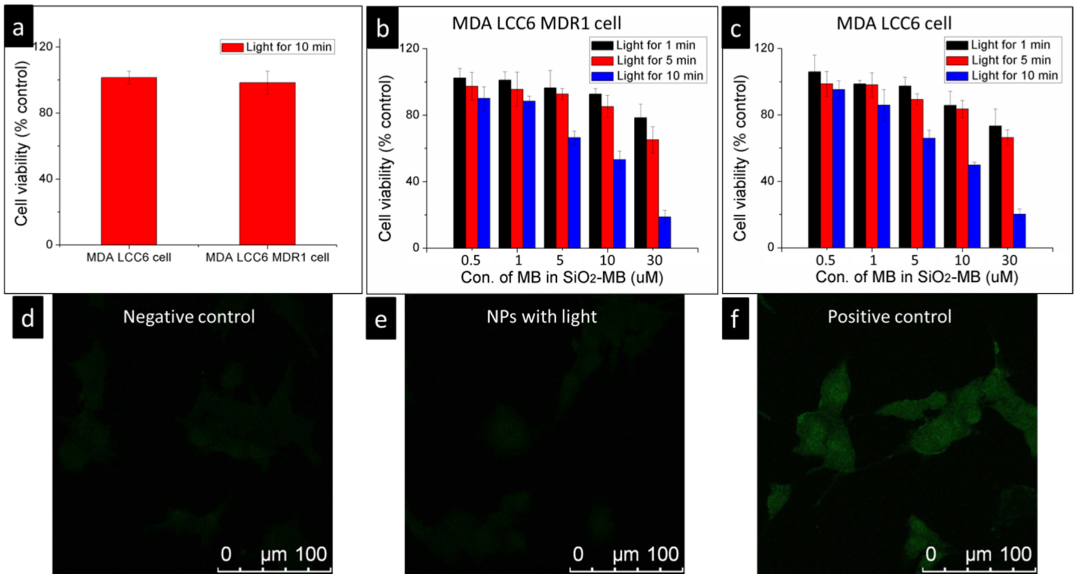

Figure S4. Cytotoxicity evaluation of SiO_2-MB carriers. (a) Cell viability after LED light irradiation for 10 min. Cell viability of (b) MDA LCC6 MDR1 cells and (c) MDA LCC6 cells as functions of MB concentration and light irradiation duration. Data were presented with mean ± standard deviation (SD) (from three independent experiments) and significantly different ($p < 0.05$) from control (analyzed by Student's t test). A typical result of Cleaved caspase-3 antibody staining of cells (d) without treating (negative control), (e) with NPs and light treating (5 μM MB, 5 min light irradiation), and (f) with Dox treating (positive control).

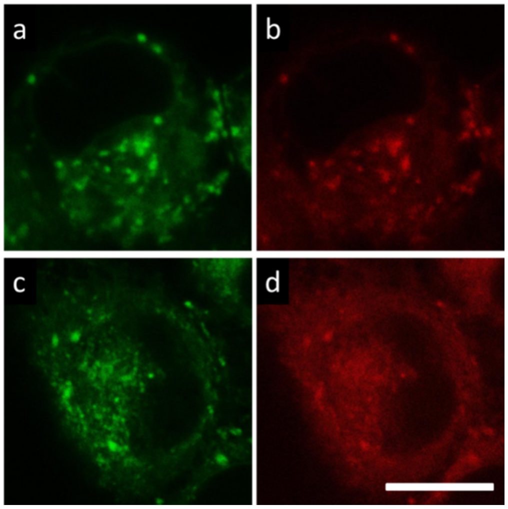

Figure S5. Observation of enhanced Dox release to cytosol by light triggering (5 min light irradiation followed by 2 h incubation, when the Dox concentration in cytosol attained equilibrium). Confocal microscopy images of the MDA LCC6 MDR1 cells treated with SiO_2(MB)-Dox NPs (a,b) without and (c,d) with light irridation. Lysosomes were stained with lysotracker (LysoTracker™ Green DND-26, with excitation and emission wavelengths of 504 and 511 nm) showing green color (a,c); Dox fluorescence signal with excitation and emission wavelengths of 470 and 570 nm were shown in red color (b,d). The scale bar is 10 μm.

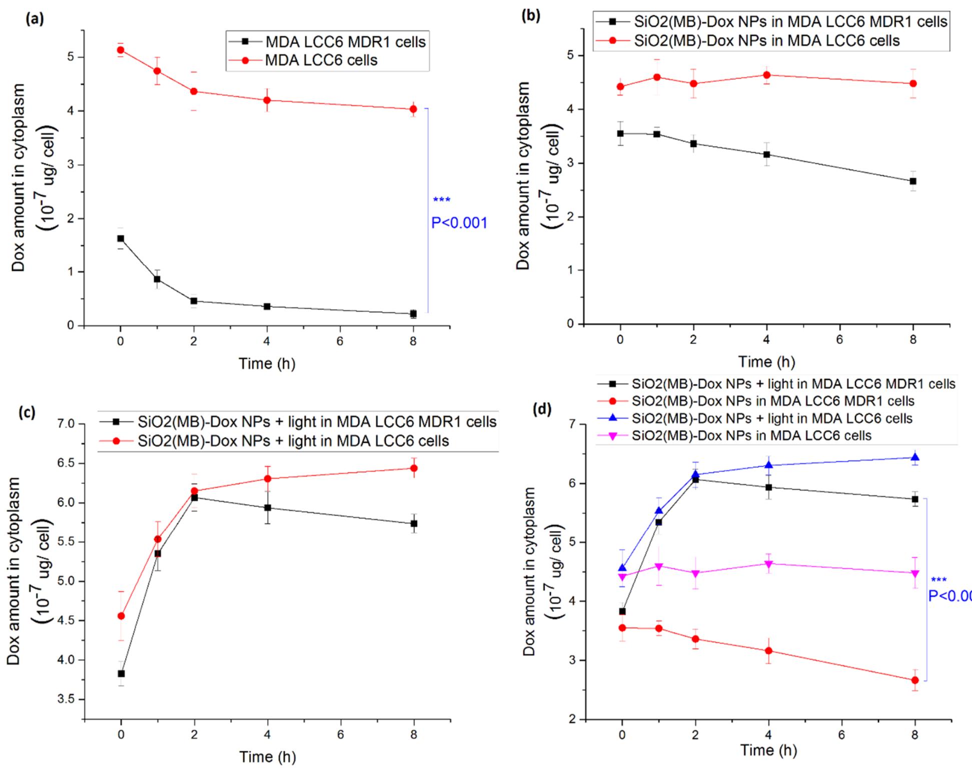

Figure S6 Multiple t-test of intracellular Dox amount in different treatment groups, performed by Prism GraphPad 7.0 software. Statistical significance was determined using the Holm-Sidak method, with alpha=0.001. (a) Intracellular Dox amount disposition of free Dox treatment groups in both MDA LCC6 cells and MDA LCC6 MDR1 cells. (b-c) Intracellular Dox amount disposition of Dox NP without/with light irridiation treatments in both MDA LCC6 cells and MDA LCC6 MDR1 cells. (d) Statistical analysis was performed between SiO_2(MB)-Dox NPs and SiO_2(MB)-Dox NPs + Light in both MDA LCC6 cells and MDA LCC6 MDR1 cells. *** represented the significant differences ($P<0.001$)

Statitical analysis results of **Figure 3**：

1) Multiple t-test was performed by Prism GraphPad 7.0 software. Statistical

significance was determined using the Holm-Sidak method, with alpha=0.001. In all time durations of free Dox treated groups in two cell lines (MDA LCC6 and MDA LCC6 MDR1), significant differences in the intracellular Dox concentration were observed (P<0.0001), indicating the MDR in MDA LCC6 MDR1 cells (**Figure S6a**).

2) When comparing the intracellular Dox concentration of NP treated groups without light irradiation in MDA LCC6 cells and MDA LCC6 MDR1 cells, despite the decreasing trend of intracellular Dox concentration in the drug resistant cells (MDA LCC6 MDR1), no significant difference appeared (P>0.001) at all time points examined (**Figure S6b**). When comparing the the intracellular Dox concentration of NP treated groups with light irradiation in MDA LCC6 cells and MDA LCC6 MDR1 cells, the difference is insignificant as well (**Figure S6c**). These results suggested reveal of MDR in the drug resistant cells. The Statistical analysis of **Figure S6d** interpreted that light irradiation significantly increased the cytosolic Dox concentration.

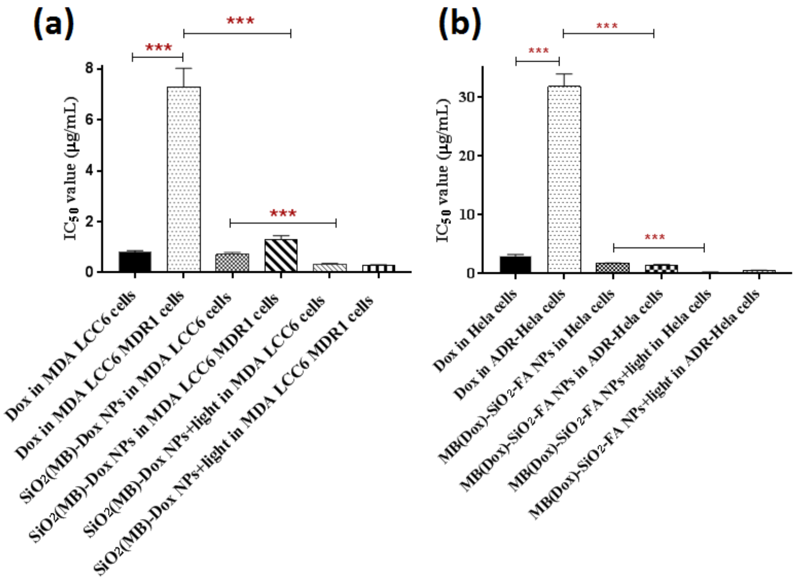

Figure S7 IC_{50} values of different treatment groups in (a) MDA LCC6 vs. MDA L6 MDR1 cells; (b) Hela vs. ADR-Hela cells. Statitical analysis were performed by Prism GraphPad 7.0 software. *** represented the significant differences ($P<0.001$).

Statitical analysis results of **Figure 4** and **Figure S8**：

Statitical analysis of IC_{50} in all treatment groups were also performed by Prism GraphPad 7.0 software (**Figure S7**).

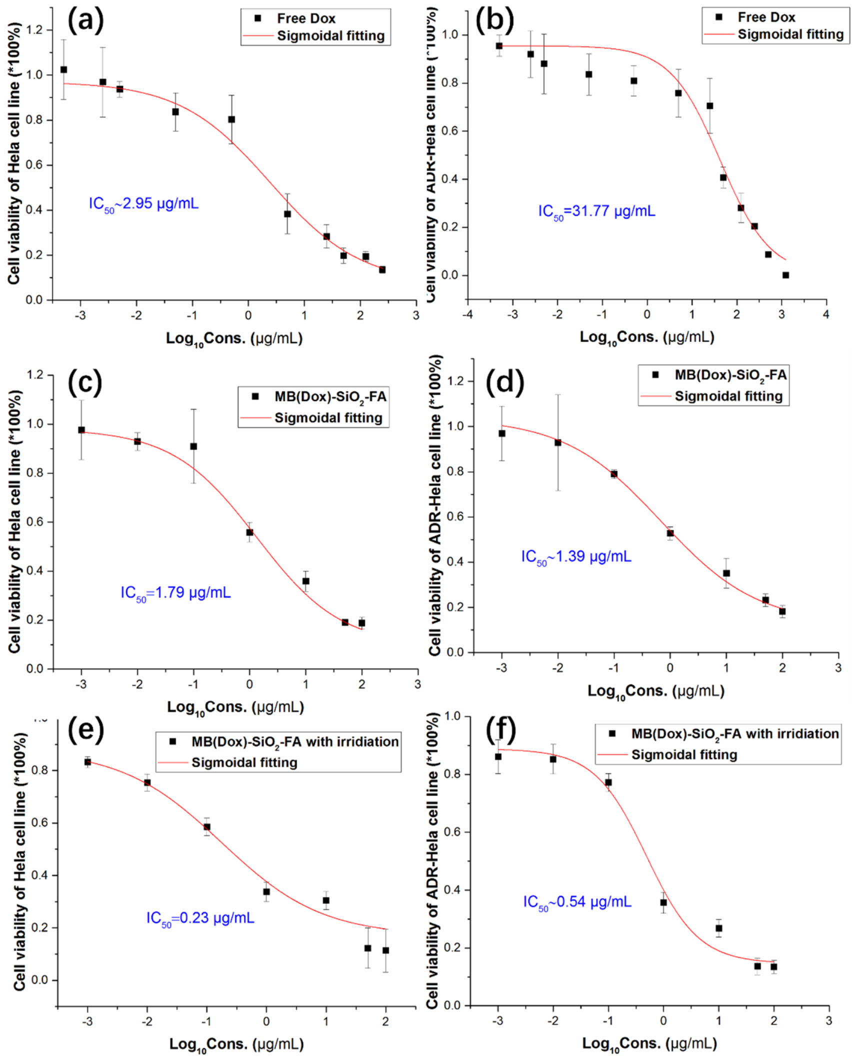

Figure S8. IC_{50} determination of free Dox and NPs treatment groups in both Hela and ADR-Hela cells. The viability of (a) Hela cells and (b) ADR-Hela cells after their exposure to pure Dox with different concentrations; (c) Hela cells and (d) ADR-Hela cells after exposure to MB(Dox)-SiO_2-FA NPs with different Dox concentrations; (e) Hela cells and (f) ADR-Hela cells after exposure to MB(Dox)-SiO_2-FA NPs with different Dox concentrations and 5 min light irradiation. Data were presented with mean ± standard deviation (SD) (from three independent experiments).

The IC_{50} of free Dox in Hela and ADR-Hela cell line were about 2.95 µg/mL (**Figure S8a**) and 31.77 µg/mL (**Figure S8b**), showing that ADR-Hela cells were about 10 times more resistant to free Dox than Hela cells. When Dox was loaded into MB-SiO_2-FA NP carrier, the drug efficacy (IC_{50}~1.79 µg/mL) in Hela cells was similar to that of free Dox (**Figure S8c vs. a**), but much lower in ADR-Hela cells than that of free Dox (showing one order of magnitude difference, **Figure S8d vs. b**). Turning on the "optical switch" in the NP system (**Figure S8e and f**) further reduced the IC_{50} values of the NP-drug in both Hela and ADR-Hela cell lines. The respective IC_{50} in Hela and ADR-Hela cells were decreased to 0.23 µg/mL and 0.54 µg/mL (**Figure S8e, f**). Both values were significantly lower than the IC_{50} of free Dox in Hela cells.

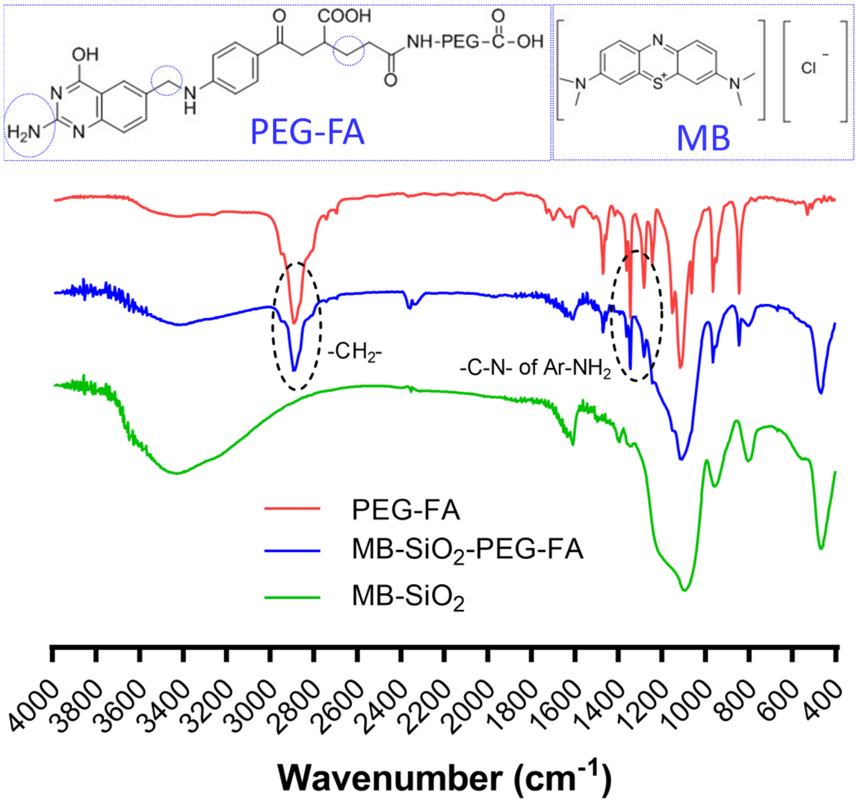

Figure S9 compares the FTIR spectra of PEG-FA, MB-SiO_2-PEG-FA NPs and MB-SiO_2 NPs.

The peaks around 2900 cm^{-1} originate from the C-H stretching vibrations in -CH_2- groups and those around 1300 cm^{-1} come from C-H stretching vibration in Ar-NH_2 groups, both of which are characteristic of PEG-FA (marked in black circles in the Figure). These peaks appear only when PEG-FA were conjugated onto the NPs surface, but not in the FTIR of MB-SiO_2 NP samples, supporting the successful surface modification of MB-SiO_2 NPs by PEG-FA.

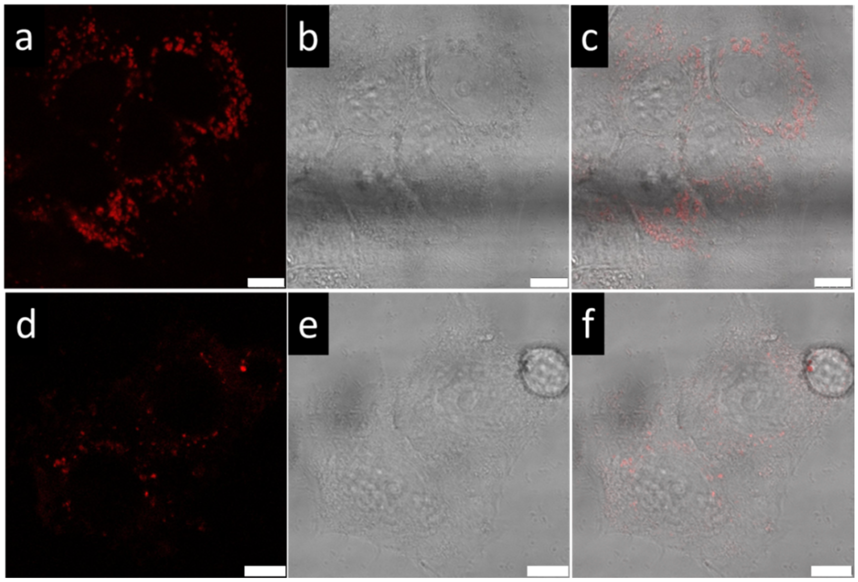

Figure S10. Cellular uptake comparison between (a,b,c) MB(Dox)-SiO_2-FA NPs and (d,e,f) MB(Dox)-SiO_2 NPs. (a,d) NPs signal images, (b,e) transmission images, (c,f) overlap images. The scale bar is 10 µm for all images.

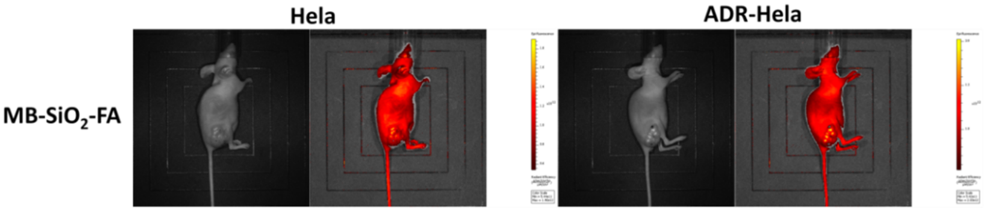

Figure S11. Targeting capability of the nanocarrier drugs as demonstrated by *in vivo* imaging of Hela and ADR-Hela tumor bearing mice. Fluorescent signal captured by IVIS Lumina Imaging System in tumor bearing mice after injection with MB-SiO_2-FA NPs for 24 hrs.

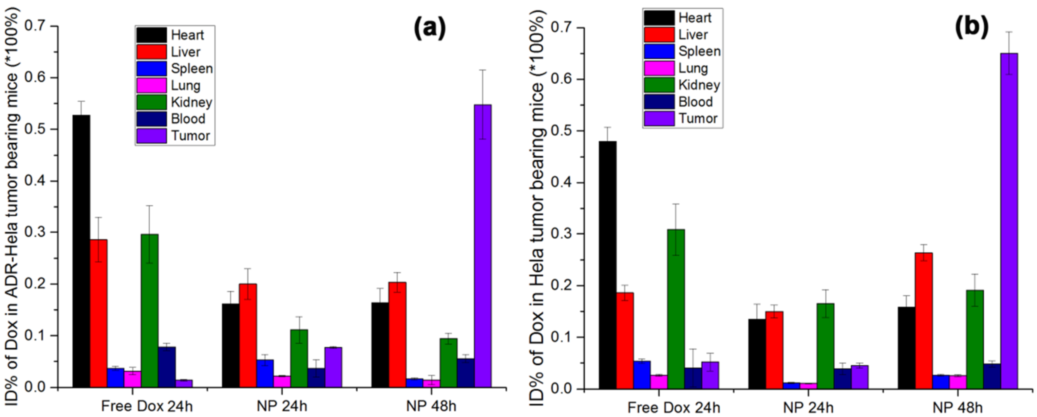

Figure S12. Evaluation of Dox in major organs and tumors in both Hela and ADR-Hela models. Fluorescence spectrophotometry analysis of Dox amounts in major organs and tumors at 24 hrs after free Dox injection, and 24/48 hrs after injection of MB(Dox)-SiO_2-FA NPs in (a) ADR-Hela tumor and (b) Hela tumor bearing mice. Data were presented with mean ± SD, n = 5.

In vivo imaging (**Figure S11**) disclosed that highlighting spots in tumor site and systemic fluorescence were observed 24 hrs after NPs administration, due to the targeting function of FA and elongated circulation promoted by PEG. Dox was not released from NPs at this time point, as suggested by the analysis of drug amount in tissues after injection (**Figure S12**). At 24 hrs after injection, small amount of Dox was released in both ADR-Hela (＜7%) and Hela (＜5%) tumor bearing mice, while over 50% of Dox was released after 48 hrs after injection in both models (**Figure S12 a and b**).

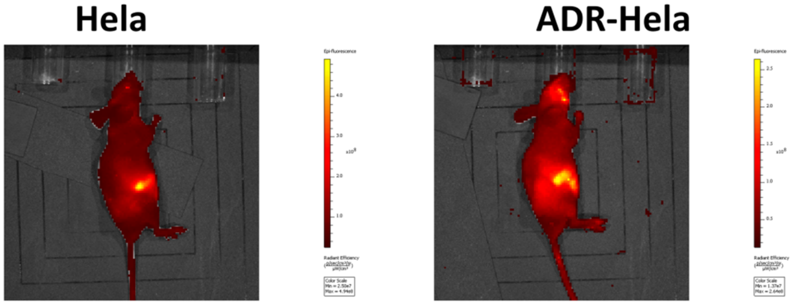

Figure S13. Targeting capability of the nanocarrier drugs as demonstrated by *in vivo* imaging of Hela and ADR-Hela tumor bearing mice. Fluorescent signal captured by IVIS Lumina Imaging System in tumor bearing mice after injection with MB-SiO_2-FA NPs for 48 hrs.

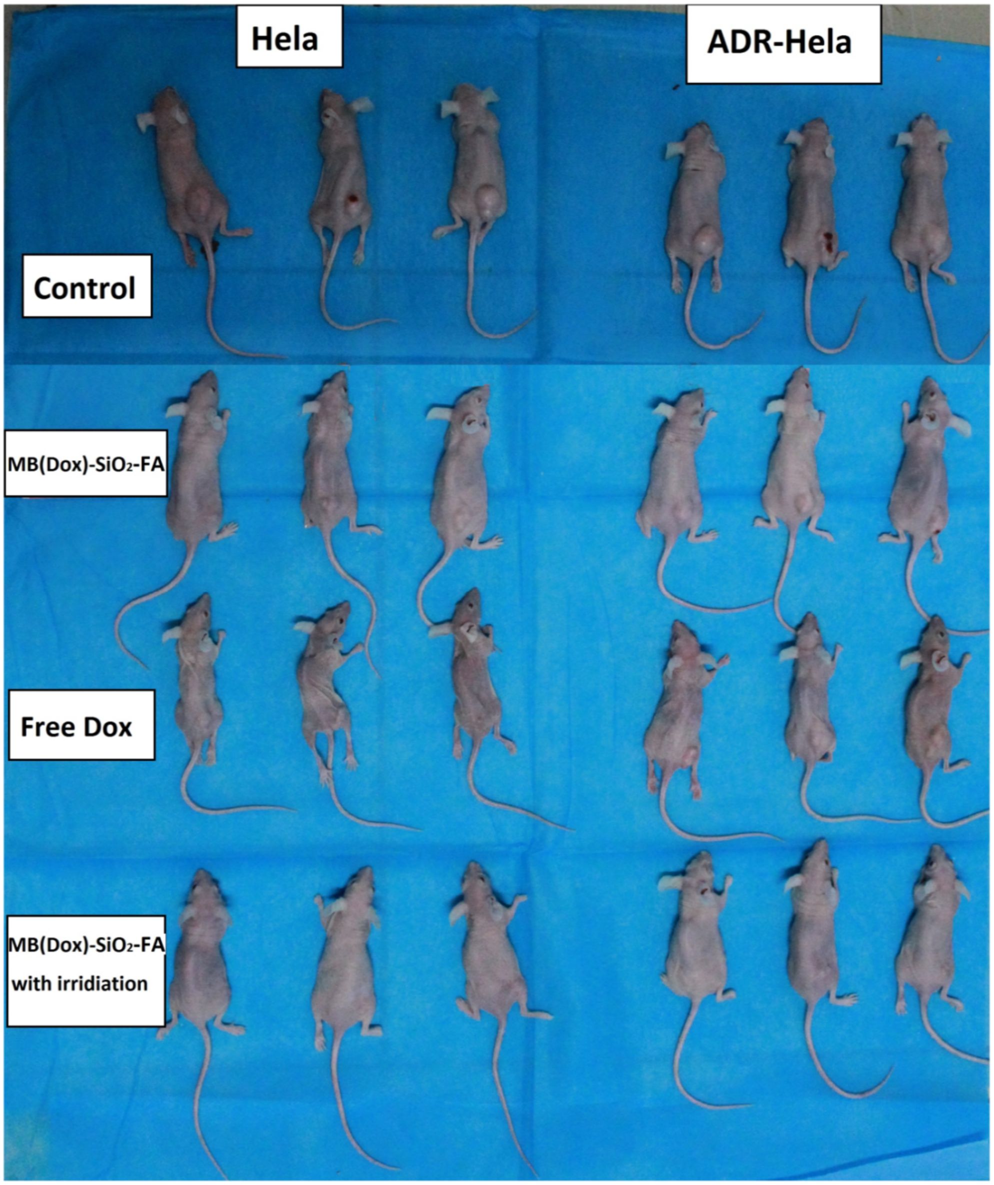

Figure S14 Photos of mice after 4 weeks treatment in different groups.

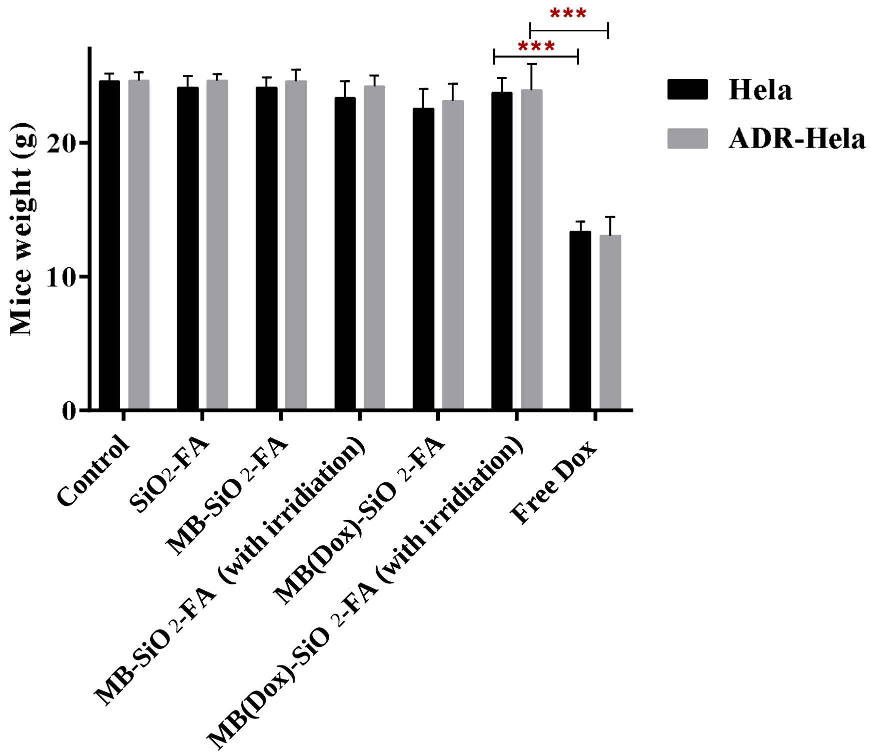

Figure S15 Body weight changes 4 weeks after administration in all treatment groups. Statitical analysis were performed by Prism GraphPad 7.0 software. *** represented the significant differences ($P<0.001$)

Only free Dox treatment groups has significant body weight decrease, indicating high systemic toxicity. All other NP treatment groups have stable body weight increase, demonstrating well biocompatiblity of NPs.

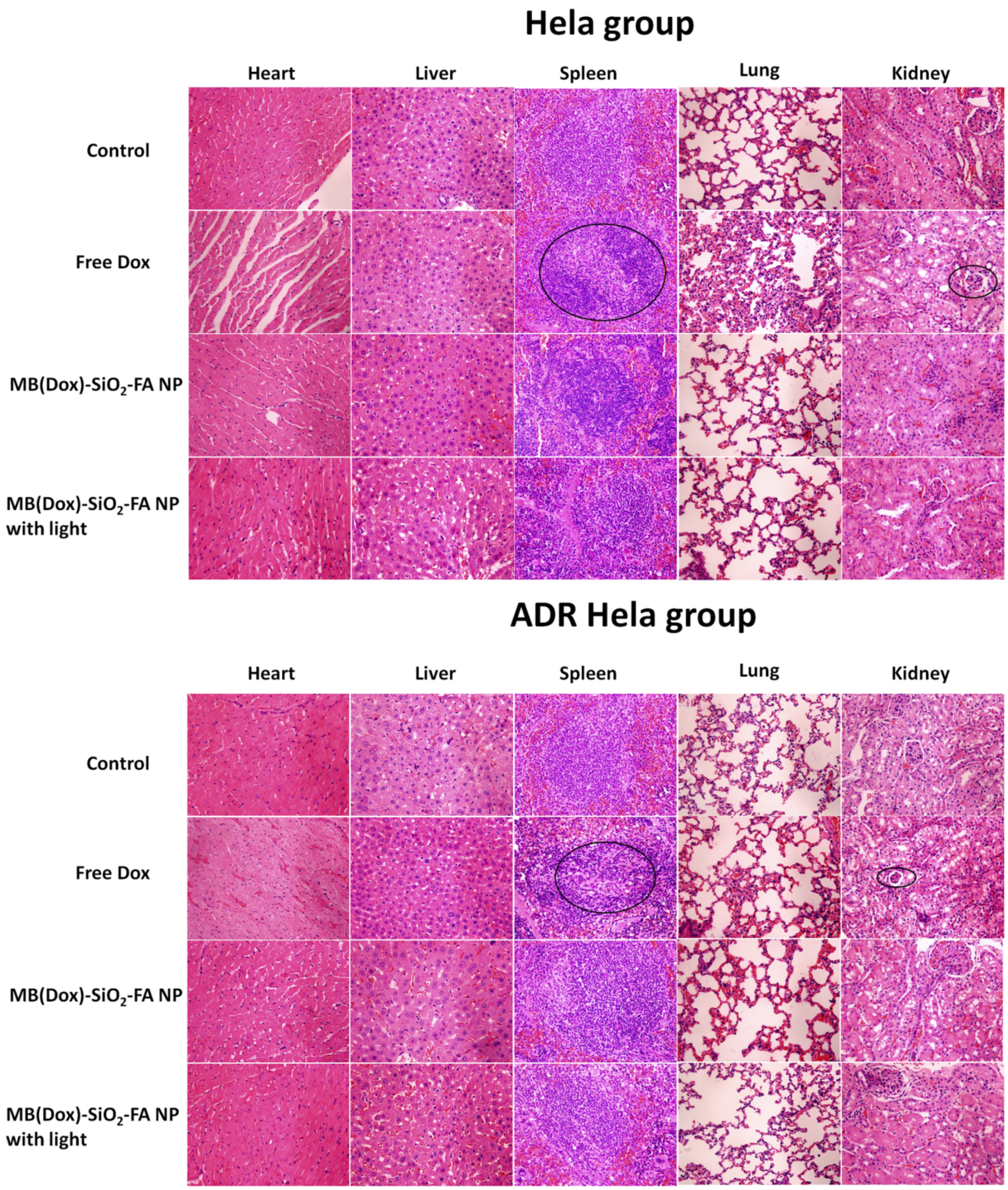

Figure S16 H&E staining used to evaluate the systemic toxicity of NPs treatment groups in both Hela and ADR-Hela cells bearing mice. H&E stained images of the heart, liver, spleen, lung and kidney in control and treatment groups. The black circle represents the lesions in the spleen and kidney area.
